# A theory-based educational intervention to pediatricians in order to improve identification and referral of maternal depression: a quasi-experimental study

**DOI:** 10.1186/1744-859X-12-37

**Published:** 2013-11-22

**Authors:** Eirini Agapidaki, Kyriakos Souliotis, Stylianos Christogiorgos, lannis Zervas, Angeliki Leonardou, Gerasimos Kolaitis, George Giannakopoulos, Christina Dimitrakaki, Yannis Tountas

**Affiliations:** 1 Center for Health Services Research, Department of Hygiene, Epidemiology and Medical Statistics, University of Athens Medical School, Alexandroupoleos st. 25, Athens 115 27, Greece; 2 Faculty of Social Sciences, University of Peloponnese, Corinth 20 100, Greece; 3 Department of Psychiatry, Athens University Medical School, Athens 124 62, Greece; 4 Department of Child Psychiatry, University of Athens Medical School, “Aghia Sophia” Children's Hospital, Athens 115 27, Greece

**Keywords:** Maternal depression, Pediatricians, Theory-based intervention

## Abstract

**Background:**

Maternal depression has a negative impact on both the mother and child's physical and mental health, as well as impairs parenting skills and pediatric health care utilization. The pediatricians' role in identification and management of maternal depression is well established. Although it can be successfully and easily treated, maternal depression remains under-recognized and under-treated. Despite the heightened emphasis, there is lack of interventions to pediatricians in order to improve detection and management of maternal depression.

**Methods:**

To address this gap, an educational intervention based on the ‘Health Belief Model’ was developed, implemented, and evaluated. The present quasi-experimental study, aimed to assess the pediatricians' knowledge, self-efficacy, beliefs, and attitudes toward maternal depression at baseline and post-intervention measurements. A total of 43 randomly selected primary care pediatricians residing in Athens completed a 59-item survey by mail in 2011. Pediatricians in the intervention group received a toolkit about the recognition and management of maternal depression, while pediatricians in the control group received a leaflet about mental health. Descriptive statistics, *t* test, chi-square, Fisher's exact test, and analysis of variance were used for the statistical analysis.

**Results:**

Post-intervention measurement revealed differences at a statistical significance level between the two groups, in the following variables: beliefs, attitudes, self- efficacy, perceived barriers, and management practices of maternal depression. Furthermore, at post-measurement, pediatricians in the intervention group demonstrated increased perceived responsibility and increased self-efficacy for detection and referral of maternal depression.

**Conclusions:**

Educational interventions to pediatricians seem to be beneficial for the improvement of the pediatricians' knowledge, self-efficacy, and attitudes regarding maternal depression. Studies using large, representative population samples are needed to provide evidence if the training interventions to pediatricians for maternal depression are translated to changes in their clinical practice and improved the patients' health outcomes.

## Background

Depression has quite high prevalence among women, especially during child-bearing years with prevalence rates ranging from 10% to 20% [1-3]. Hormonal, psychological, and social factors in the pre-natal and post-natal periods, as well as in early child-rearing years, have been strongly linked to the onset of depression [4]. Maternal depression is related to the child's physical, mental, and social health. It may interfere with mother-infant interaction [5-10], and it may impede child neurophysiological [11-13] cognitive, and socio-emotional development [14-17]. The mothers' depression has also been linked to anxiety, depression [18,19], attention deficit/hyperactivity disorder, and externalizing problems among their offspring [20].

Previous studies pointed out that women facing depression in the perinatal period are at increased risk for pre-term delivery and low birth weight of offspring compared to non-depressed mothers [21]. Maternal depression is also associated with breastfeeding cessation, less healthful feeding and sleeping practices in infants [14,22], and also failure to thrive [23]. There is evidence that maternal depression results in poor physical child health outcomes by affecting parenting behaviors and skills [24]. Underuse of child health preventive practices and overuse of pediatric emergency services have also been reported in studies of depressed mothers [21,25-28].

Pediatricians are the health care providers with the most frequent contacts with women of child-bearing age and therefore could play a key role in detecting maternal depression during the well-child visits [29-32]. Despite this, pediatricians do not seem to sufficiently address the issue [33]. Delays in detection of depression set the mother and the child in enormous risk and deprive the opportunity for the mother to receive appropriate care from health care services or participate in a mental health promotion intervention. The early recognition and management of maternal depression are implied by the European policy framework that includes ‘support parenting and the early years of life’ as a major action area [34].

Barriers exist both at organizational and individual levels. The most common organizational barriers for maternal depression screening and management include (1) work overload and time restrictions, (2) absence of interdisciplinary team work, (3) lack of relevant health insurance coverage, (4) poor cooperation and collaboration between general and mental health sectors, and (5) inadequate community mental health services [30,35-37]. Facilitating factors include the establishment of supportive policies for family-oriented health care and the integration of general health and mental health services [33,38]. The most frequently reported barriers at the pediatrician level include (1) reduced awareness and limited skills regarding basic aspects of maternal depression (clinical manifestation, impact, screening, and management practices), (2) perceived roles and responsibilities, (3) poor perceived self-efficacy, (4) fear of stigmatizing mothers, and (5) previous negative experiences in communicating with mental health professionals [39,40]. The importance of addressing the above individual level's obstacles is of high a priority since they are common barriers at both public and private pediatric settings and they can be more easily modified compared to the organizational ones [1,37,41,42]. Educational interventions and training in the use of screening tools, semi-structured clinical interviews, and counseling skills seem to be beneficial and can increase the health professionals' self-efficacy and early recognition and management of maternal depression [43,44]. The European Forum for Primary Care [45] suggests that a strategy indicating positive results in recognition and management of depression is the training for primary health care professionals in depression screening tools.

It should be stressed that theory is a crucial aspect of all health promotion learning interventions. Theory provides a framework to reason how and why people alter their behavior [46]. It also aids the evaluation of the program's effectiveness [47]. The absence of theory cannot ensure that all the variables which influence the problem are targeted [48].

The present study aimed to examine the impact of a theory-based educational intervention to pediatricians, in order to improve early identification and management of maternal depression. It was hypothesized that the intervention would manage to (1) increase the pediatricians' knowledge about maternal depression and its effect on children health, mental health, and well-being, (2) change the pediatricians' beliefs about the severity and impact of maternal depression, (3) change the pediatricians' attitudes toward their responsibility for the identification and follow-up of mothers with depression, and (4) increase the pediatricians' perceived self-efficacy toward the identification and referral of mothers with depression.

## Methods

### Participants and procedures

The intervention study was conducted in collaboration with the Centre of Health Services Research, Medical School of Athens and approved by the institutional Review Board. A quasi-experimental non-equivalent control group design with pre-test-post-test measurements was carried out. Participants were primary health care pediatricians, practicing in community pediatric settings in Athens. Pediatricians were excluded if they had previous training on maternal depression or were practicing in a non-primary health care setting.

Pediatricians who met the aforementioned inclusion criteria were identified through the Medical Association of Athens. For the purpose of the study, four different geographical regions were randomly selected. From the 84 eligible pediatricians, 60 were randomly selected by using a random number table. The selected pediatricians were informed about the purpose and procedures of the study. A total of 43 participants (i.e., response rate of 72%) accepted to participate, signed the informed consent form, and completed the baseline assessment. After the baseline measurement, assignment to either intervention or comparison condition was conducted by matching. The matching took place at the pediatrician's level (individual), taking into account demographic variables, prior related training, and self-rated skills on detection and management of maternal depression. To avoid potential bias, a double concealed procedure was implemented. Neither participants nor researchers were aware about the assignment condition (i.e., intervention or comparison).

### Measurements

The questionnaires administered at baseline and post-intervention measurements were (a) the ‘Maternal Depression Management Inventory’ [1], (b) a socio-demographic scale, and (c) a knowledge checklist for depressive symptoms. For the purpose of the study, the ‘Maternal Depression Management Inventory’ [1] was forward and back translated and culturally adapted, according to the ‘International Society for Pharmacoeconomics and Outcomes Research’ guidelines [49].

The ‘Maternal Depression Management Inventory’ is a valid and reliable self-reported instrument of 59 items in total including demographics. It assesses knowledge, beliefs, attitudes, self-efficacy, current practices, and perceived barriers of pediatricians regarding detection and management of maternal depression. Thirty-five items out of 59 are measuring knowledge, beliefs, attitudes, self-efficacy, current practices, and perceived barriers of pediatricians regarding detection and management of maternal depression [1]. It uses a six-point Likert-type scale for the 35 out of the 59 questions in which pediatricians can indicate the extent of their agreement with each statement (1 = strongly agree, 2 = agree, 3 = somewhat agree, 4 = somewhat disagree, 5 = disagree, and 6 = strongly disagree; possible responses for the items assessing current detection and management practices are ‘Daily,’ ‘Weekly,’ ‘Monthly,’ ‘Rarely,’ ‘Never’). In the majority of questions, higher scores indicated better knowledge, beliefs, attitudes, increased self-efficacy, improved current practices, and decreased perceived barriers of pediatricians regarding the detection and management of maternal depression. There are also some reverse scored items.

The ‘Maternal Depression Management Inventory’ assesses knowledge about depression through the question ‘I am familiar with the DSM-IV criteria for depression.’ We hypothesized that even if pediatricians were not familiar with the DSM-IV, they could however have knowledge on the depressive symptomatology. Therefore, we developed a checklist to evaluate their knowledge about depressive symptoms. Both the Greek version of the ‘Maternal Depression Management Inventory’ (35 items, *a* = 0.82) and the depression symptoms checklist were found to be reliable (20 items, *a* = 0.81). The socio-demographic scale contained questions about gender, age, years of practicing medicine, years of practicing pediatrics, past training in mental health, maternal mental health issues, etc.

### Intervention description

The educational intervention was based on the conceptual framework suggested by Leiferman et al. [1]. They used an integrated approach based on the Health Belief Model [46] and Social Ecological theory [50] in order to investigate the determinants of maternal depression management practices in pediatric primary health care settings. They found that the Health Belief Model provides a useful conceptual framework for future interventions aiming to facilitate changes at pediatricians' management practices toward maternal depression. Approximately 1 month after the baseline measurement, pediatricians in the intervention group received a toolkit for the recognition and referral of maternal depression by mail.

The toolkit was developed according to the Health Belief Model and consisted of a booklet with information about the frequency, severity, impact, and symptoms of maternal depression; guidelines for screening, the Patient-Health Questionnaire-2 [51,52] with an instruction manual, case studies, and factsheets for detection and referral. The booklet aimed to address the barriers at the provider's level according to the Health Belief Model (e.g., perceived impact of maternal depression, perceived responsibility for detection and follow-up, barriers, and self-efficacy). The toolkit also included (a) a list with available local community mental health services for referral, (b) posters and information leaflets for maternal depression which was placed in pediatric waiting room in order to motivate and facilitate mothers to discuss about depression, and (c) a screening reminder, as a ‘cue to action.’ The screening reminder was a flow chart with the steps needed to be followed for detection and referral of maternal depression. The flowchart was suggested to be placed on the pediatrician's office in order to remind the process that should be followed. Participants in the comparison group received only an information leaflet about general mental health issues.

### Statistical analysis

Quantitative variables were expressed as mean values or frequencies. Internal consistency reliability was determined by the Cronbach's α coefficient. Reliability equal to or greater than 0.70 was considered acceptable. Mean differences were applied in order to examine any differences between groups after assignment. Independent samples Student's *t* tests were used for the comparison of mean values between the control and the intervention group. For the comparison of proportions chi-square and Fisher's exact tests were used. Repeated measurements analysis of variance (ANOVA) was adopted to evaluate the changes observed between comparison and intervention group over the follow-up period. Due to the skewed distributions for repeated measurements analysis of variance, the ranks of the variables were used. All reported *p* values are two-tailed. Statistical significance was set at *p* < 0.05, and analyses were conducted using SPSS statistical software (version 18.0).

## Results

Sample consisted of 43 subjects (21 to the comparison group and 22 to the intervention group). Sample characteristics for both groups are presented in Table 1. Analyses did not reveal any statistically significant differences between the two groups prior to the intervention (Table 1) in terms of demographics, prior related training, and self-rated skills (*p* > 0.05).

**Table 1:** Sample demographics by group

	**Total sample (*N*** **= 43)**	**Comparison group (*N*** **= 21)**	**Intervention group (*N*** **= 22)**
	***N *** **(%)**	***N *** **(%)**	***N *** **(%)**
Gender			
Men	15 (34.9)	7 (33.3)	8 (36.4)
Age, mean (SD)	53.8 (6.9)	53.7 (6.7)	53.9 (7.3)
Years practicing medicine			
≤ 15	6 (14)	2 (9.5)	4 (18.2)
> 15	37 (86)	19 (90.5)	18 (81.8)
Years practicing pediatrics			
≤ 15	19 (44.2)	8 (38.1)	11 (50.0)
> 15	24 (55.8)	13 (61.9)	11 (50.0)
Setting of practice			
Private office	38 (88.4)	19 (90.5)	19 (86.4)
Private office and clinic	5 (11.6)	2 (9.5)	3 (13.6)
Years in current setting			
≤ 10	18 (41.9)	9 (42.9)	9 (40.9)
> 10	25 (58.1)	12 (57.1)	13 (59.1)
Attending mental health courses in			
Undergraduate studies	40 (93)	19 (90.5)	21 (95.5)
Post-graduate studies	3 (7)	1 (4.8)	2 (9.1)
Training, seminar, workshop	3 (7)	0 (0)	3 (13.6)
Preconference workshop	9 (20.9)	3 (14.3)	6 (27.3)
Other	1 (2.3)	1 (4.8)	0 (0)
Number of mothers visiting your setting per month			
20-30	30 (69.8)	16 (76.2)	14 (63.6)
50	13 (30.2)	5 (23.8)	8 (36.4)
Do you have a contractual agreement with a public health insurance fund?	35 (81.4)	16 (76.2)	19 (86.4)
Relatives/personal friends with a mental disease	20 (46.5)	11 (52.4)	12 (54.5)
‘How would you rate your professional training in detecting maternal depression’			
Never received training	22 (51.2)	13 (61.9)	9 (40.9)
Poor	14 (32.6)	7 (33.3)	7 (31.8)
Good	7 (16.3)	1 (4.8)	6 (27.3)
‘How would you rate your professional training in treating maternal depression’			
Never received training	22 (51.2)	13 (61.9)	9 (40.9)
Poor	15 (34.9)	7 (33.3)	8 (36.4)
Good	6 (14)	1 (4.8)	5 (22.7)

Differences in knowledge scores toward maternal depression after the intervention for the comparison and intervention group are presented in Table 2. Higher mean values indicated greater knowledge. Participants in the intervention group increased their knowledge score on depression symptoms after the intervention significantly more (*p* < 0.001) than those in the comparison group (*p* > 0.05).

**Table 2:** Changes in knowledge status between comparison and intervention group at pre- and post-assessments

	**Pre**	**Post**	**Change**		
	**Mean (SD)**	**Mean (SD)**	**Mean (SD)**	***P*** **	***P***^**‡**^
I am familiar with the DSM-IV criteria for depression					
Comparison group	1.9 (0.9)	1.9 (0.4)	0.0 (0.9)	0.957	<0.001
Intervention group	2.1 (1.2)	3.5 (0.7)	1.4 (1.4)	0.003	
P*	0.783	<0.001			
Depression symptoms scale					
Comparison group	65.0 (14.1)	89.7 (6.1)	24.7 (13.8)	<0.001	0.591
Intervention group	71.6 (16.1)	96.0 (6.8)	24.4 (18.1)	<0.001	
P*	0.162	0.002			

*p value for differences between the two groups; **p value for changes among pre- and post-measures; ^‡^repeated measurements ANOVA, *p* value for interaction effect of time with group.

Changes in self-efficacy for the identification and referral of maternal depression after the intervention for the comparison and intervention group are presented in Table 3.

**Table 3:** Self-efficacy for identification and management between comparison and intervention group at pre- and post-assessments

	**Pre**	**Post**	**Change**		
	**Mean (SD)**	**Mean (SD)**	**Mean (SD)**	***P*** **	***P***^**‡**^
I feel confident in my ability to identify maternal depression.					
Comparison group	2.0 (0.7)	1.9 (0.3)	−0.1 (0.7)	0.763	<0.001
Intervention group	2.5 (1.1)	3.3 (0.6)	0.8 (1.2)	0.008	
P*	0.125	<0.001			
I feel confident in my ability to treat (provide referral, counseling) maternal depression					
Comparison group	2.2 (0.7)	1.9 (0.3)	−0.3 (0.9)	0.083	<0.001
Intervention group	2.3 (0.9)	3 (0.5)	0.7 (1.2)	0.016	
P*	0.885	<0.001			
I feel comfortable talking about depression with patients.					
Comparison group	3.6 (0.9)	3.0 (0.8)	−0.6 (1.0)	0.009	0.035
Intervention group	3.5 (0.9)	3.5 (0.6)	0.0 (1.0)	1.000	
P*	0.733	0.022			
P*	0.445	0.008			
I am comfortable contacting a mental health professional to consult about a patient.					
Comparison group	4.4 (0.9)	4.7 (0.7)	0.3 (1.1)	0.813	0.741
Intervention group	4.5 (0.7)	4.5 (0.9)	0.0 (1.1)	0.817	
P*	0.863	0.779			

*p value for differences between the two groups; **p value for changes among pre- and post-measures; ^‡^repeated measurements ANOVA, *p* value for interaction effect of time with group.

Higher mean values indicated greater agreement. Prior to the intervention, no significant differences were found between the two groups regarding self-efficacy for the identification and management of maternal depression. Post-intervention, participants in the intervention group reported increased confidence toward detection of maternal depression compared to participants in the comparison group (*p* < 0.001). There was a significant increase of the confidence levels in detecting maternal depression only for the intervention group.

Changes in the pediatricians' beliefs toward the impact of maternal depression as well as their perceived responsibilities for detection and follow-up for both groups are presented in Table 4, with higher mean values indicating greater agreement.

**Table 4:** Changes in beliefs toward the impact and detection/follow-up responsibility for both groups

	**Pre**	**Post**	**Change**		
	**Mean (SD)**	**Mean (SD)**	**Mean (SD)**	***P*** **	***P***^**‡**^
Depressed mothers provide more inconsistent care to their children than non-depressed mothers.					
Comparison group	5 (0.9)	4.8 (0.6)	−0.2 (0.9)	0.261	0.974
Intervention group	5.2 (0.8)	5 (0.7)	−0.2 (1.2)	0.397	
P*	0.599	0.480			
Maternal depression often goes away without treatment.					
Comparison group	3.4 (1.1)	3.4 (0.9)	0 (1.4)	0.874	0.097
Intervention group	3.3 (1.5)	2.5 (0.9)	−0.8 (1.8)	0.073	
P*	0.795	0.001			
It is normal for mothers of young children to feel depressed.					
Comparison group	3.5 (1.2)	4 (0.7)	0.5 (1.2)	0.061	0.001
Intervention group	3.6 (1.1)	2.7 (1)	−0.9 (1.4)	0.010	
P*	0.850	<0.001			
Recognizing maternal depression is my responsibility.					
Comparison group	1.7 (0.6)	2.1 (0.6)	0.4 (0.8)	0.014	<0.001
Intervention group	1.8 (1)	4.4 (0.7)	2.6 (1)	<0.001	
P*	0.536	<0.001			
Treating (e.g., counseling, provide referral) maternal depression in my patients or their mothers is my responsibility.					
Comparison group	1.9 (0.6)	2.2 (0.7)	0.3 (0.9)	0.057	<0.001
Intervention group	2.1 (0.6)	3.9 (0.8)	1.8 (1)	<0.001	
P*	0.140	<0.001			
It is my responsibility to refer depressed mothers for further mental health treatment.					
Comparison group	3.2 (1.1)	3.1 (1)	−0.1 (1.4)	0.880	0.009
Intervention group	3.5 (1)	4.5 (0.7)	1 (0.9)	<0.001	
P*	0.282	<0.001			
It is not my responsibility to follow up after making a referral to a mental health specialist.					
Comparison group	4.7 (0.7)	4.9 (0.5)	0.2 (0.8)	0.296	0.001
Intervention group	4.7 (0.8)	3.6 (1)	−1.1 (1.5)	0.001	
P*	0.956	<0.001			
I do not have time to follow up with the patient after making a referral.					
Comparison group	4 (0.9)	4.9 (0.4)	0.9 (0.9)	<0.001	<0.001
Intervention group	3.8 (0.8)	3.3 (0.8)	−0.5 (1.1)	0.053	
P*	0.370	<0.001			

*p value for differences between the two groups; **p value for changes among pre- and post-measures; ^‡^repeated measurements ANOVA, *p* value for interaction effect of time with group.

Post-intervention measurement revealed that participants of the intervention group changed significantly more their beliefs about the impact of maternal depression and their beliefs for the detection/follow-up responsibility. The overall change at pre- and post-assessments between groups was significantly different (*p* = 0.001).

Changes in current management practices about detection, management, and follow-up of maternal depression at post-measurements for both groups are presented in Table 5.

**Table 5:** Changes in current management practices between comparison and intervention group at pre- and post-assessments

	**Pre**	**Post**	**Change**		
	**Mean (SD)**	**Mean (SD)**	**Mean (SD)**	***P*** **	***P***^**‡**^
How often do assess for maternal depression among mothers demonstrating depressive symptomatology during their healthcare visit?					
Intervention group	4.8 (0.4)	5.0 (0.2)	0.2 (0.4)	0.847	0.787
Comparison group	4.6 (0.6)	4.7(0.5)	0.3 (0.8)	0.850	
P*	0.132	0.022			
How often do you provide counseling for maternal depression in your daily practice;					
Comparison group	1.8 (0.5)	1.5 (0.5)	−0.3 (0.6)	0.503	0.350
Intervention group	2 (0.4)	1.8 (0.4)	−0.2 (0.5)	0.513	
P*	0.272	0.042			
How often do you refer a patient for treatment of maternal depression;					
Comparison group	2 (0.4)	2 (0.1)	0.0 (0.4)	0.417	0.258
Intervention group	2 (0.3)	1.9 (0.3)	−0.1 (0.4)	0.428	
P*	0.647	0.162			

*p value for differences between the two groups; **p value for changes among pre- and post-measures; ^‡^repeated measurements ANOVA, *p* value for interaction effect of time with group.

Higher values indicated more frequent implementation of detection and management practices. At baseline, there were no differences between groups. Even though after the intervention participants in the intervention group appear to implement assessment (*p* = 0.022) and counseling practices (*p* = 0.042) significantly more than pediatricians in the comparison group, this does not suggest that there was an actual change in detection and management practices because the differences between two groups at follow-up measurements were too slight. Moreover, the overall change in assessment and management practices (counseling and/or referral) was not significantly different between the two study groups.

## Discussion

The present study aimed at examining the impact of a theory-based educational intervention for pediatricians in order to improve the detection and referral of maternal depression. At post-intervention measurements, pediatricians in the intervention group increased their knowledge on depressive symptoms, held more positive attitudes toward their role in the identification of maternal depression, and increased their self-efficacy about the detection of maternal depression, the referral to mental health specialists, and the follow-up of referred mothers, significantly more than pediatricians in the comparison group did.

Results suggest that a brief theory-based intervention can be beneficial at overcoming the belief that detection of maternal depression is not part of the pediatrician' role [37,53]. Previous research on the topic showed that self-efficacy is strongly linked to the actual behavior of detection and management practices [1]. Papadopoulou et al. [54] found that educational interventions to primary health care professionals could indeed increase self-efficacy and perceived skills toward management of post-partum depression. Self-efficacy has been shown to predict consequently the short- and long-term health-related behavioral changes [55]. In the context of the Health Belief Model, self-efficacy is understood as a moderator of perceived personal barriers [46].

The Health Belief Model also defines that a person has increased possibilities to take action if he/she is exposed to ‘cues to action’ and feel confident to carry out the action by overcoming the personal barriers [46]. Previous research showed that educational efforts in psychosocial issues can modify pediatricians' perceived barriers at individual level (e.g., self-efficacy toward detection and management, knowledge) [53] and may contribute to the change of their practice behaviors [30,56]. Head and her colleagues [56] also found that pediatricians who are more educated on maternal psychosocial issues have decreased possibilities to report self-perceived barriers. The variables targeted by the Health Belief Model not only provide a useful conceptual framework in order to understand pediatricians' beliefs and attitudes toward maternal depression [1] but also appear to deliver a useful tool to modify them.

Destigmatization and misconceptions regarding maternal depression were main variables targeted in the present educational efforts. The toolkit given to the intervention group also included an information leaflet designed to be placed in the pediatric waiting room to facilitate mothers to discuss about potential emotional problems, probably creating a non-threatening environment toward disclosure. Stigmatization of mental disorders and maternal depression is a common barrier [57,58] for both the mother and the physician, which often deprives the opportunity for the mother to seek for help.

In contrast to our hypothesis, there were no statistically significant changes between groups at their detection and management practices, as measured 1 month after the intervention, although tiny significant differences within groups observed. Similar results - i.e., changes on knowledge, subjective responsibility, and self-efficacy for detection and referral but not changes in everyday clinical practices (e.g., improved screening rates) - have been reported in most relevant educational efforts aiming to incorporate a mental health service in primary health care settings [59,60]. Mishina and his colleagues [59] found that their educational intervention to pediatricians did not improve the screening rates for maternal depression. They suggested that one of the main barriers for detection of maternal depression is stigmatization, especially in the cases of mothers who do not have observable depressive symptoms but they score positive in screening. They advocated that comprehensive continuing education with feedback provision and empowerment aspects is needed to enhance the pediatricians' knowledge to discuss about depressive symptoms. Olson and her colleagues [42] evaluate the feasibility of screening for maternal depression in a private community pediatric setting. They found that screening is feasible and could be sustained in primary pediatric settings; they emphasized that the integration of screening forms into daily practice contribute to the achievement of higher screening rates, while the main barrier for screening decline was staffing changes.

It is argued that low maternal depression screening rates in pediatric settings may indicate the significance of systemic barriers - mainly, the fragmented collaboration with community mental health services and the perceived low quality of such services by the pediatrician. Perceived low quality and low availability of community mental health services are associated to lower pediatricians' training opportunities on maternal depression or continuing education on maternal mental health and to their higher reporting of barriers [35]. The available web-based educational programs are limited worldwide, although web-based training could serve as an easily accessible and low-cost tool for the training needs of physicians regarding depression [61]. Pediatricians need brief, focused, and up-to-date training programs providing specific and easy to use tools which can be easily incorporated into daily practice [38]. Future research efforts should explore the effect of providing brief and targeted interventions to pediatricians on overcoming such systemic barriers.

Results in the present study should be considered in the light of the following limitations. The impact of the intervention is challenged by the small sample size. In addition, the study failed to implement full randomization, generating further validity problems. Although there were no differences at a statistical significant level before intervention between two groups, differences such as motivation may have a confound effect on the results. Another limitation of the current study is that a single item analysis was performed that probably permits no more than a correspondingly small degree of differentiation.

## Conclusions

Theory-based interventions could provide useful insights for the factors associated with the integration of maternal depression screening into primary pediatric setting. Such findings may be useful for the development of more targeted and effective interventions. On the other hand, changes in the pediatricians' knowledge, attitudes, beliefs, and self-efficacy did not lead to changes in daily clinical practice and improved health outcomes for the mother and child. We believe that future research studies using large, representative samples, investigating if the educational interventions to pediatricians may result in changes in clinical practice and improved patient's health outcomes are needed in order to provide evidence about the effectiveness of training interventions to pediatricians for the prevention of maternal depression.

## Competing interests

The authors declared that they have not competing interests.

## Authors’ contributions

EA worked on the conceptualization, design, interpretation, preparation of manuscript, editing, and revising. KS helped in the design, interpretation, comments on first draft, revision, and editing. SC helped in the design, interpretation, editing, and revising. IZ took part in the design, interpretation, supervision of the data collection, and editing. AL joined in the design, data collection, and editing. GK helped produce the design, interpretation, and editing. GG helped prepare the design, comments on first draft, revising, and editing. CD helped generate the design, comments on first draft, and editing. YT helped form the overall coordination and comments on first draft. All authors have read and approved the final manuscript.
